# A case–control study on blood vessel morphology, hemodynamic parameters, and rupture status of posterior communicating artery aneurysms

**DOI:** 10.3389/fneur.2026.1799930

**Published:** 2026-05-19

**Authors:** Yanqing Yin, Jianghua Zhang, Zixiong Huang, Zhuangbin Liao, Heng Lin, Jie Li, Xiaoao Long, Jincheng Huang

**Affiliations:** Department of Neurosurgery, Affiliated Hospital of Guangdong Medical University, Zhanjiang, China

**Keywords:** hemodynamics, indicators, morphology, posterior communicating artery aneurysm, risk factor, rupture

## Abstract

**Background:**

Individual assessment of intracranial aneurysm (IA) rupture risk is difficult; a deeper understanding of the factors that precipitate rupture is crucial to advance clinical decision-making. This study aims to analyze risk factors for rupture in posterior communicating artery (PComA) aneurysms, focusing on the influence of vascular morphology and hemodynamic parameters. Specifically, it examined demographic and clinical characteristics, vessel morphology, and hemodynamic factors, aiming to identify predictors of aneurysm rupture and inform preventive strategies.

**Methods:**

A retrospective analysis was conducted using clinical data of 362 patients with ruptured and unruptured PComA aneurysms from January 2020 to December 2024. Statistical analyses assessed differences between groups, associations between demographic and clinical characteristics, vessel morphology, and hemodynamic parameters and rupture status, and predictive factors for aneurysm rupture.

**Results:**

Two hundred and twenty-four patients with ruptured PComA aneurysms (50 male, 174 female) in the ruptured PComA aneurysm group were compared with 138 patients with unruptured PComA aneurysms (30 male, 108 female) in the unruptured PComA aneurysm group. Findings revealed significant differences in smoking status, educational experience, blood pressure, aneurysm size, shape feature, aneurysm inclination angle, size ratio (SR),wall shear stress (WSS), and flow velocity (FV) (*p* < 0.05). Smoking status, systolic pressure and diastolic pressure, aneurysm size, irregular shapes, aneurysm inclination angle and SR in the ruptured PComA aneurysm group were higher than those in the unruptured PComA aneurysm group (*p* < 0.05). And educational experience, WSS, and FV in the ruptured PComA aneurysm group were lower than those in the unruptured PComA aneurysm group (*p* < 0.05).

**Conclusion:**

In hypertensive smokers with lower educational experience, several factors may be associated with a higher risk of PComA aneurysm rupture: larger aneurysm size, irregular shape, higher inclination angle and SR, as well as lower WSS and FV. However, the relative contributions of morphological and hemodynamic factors, their possible correlations, and the ability of these markers to predict rupture risk require external validation. Therefore, larger prospective studies, multi-center investigations, and follow-up of ethnically diverse cohorts of patients with unruptured PComA aneurysms are warranted.

## 1 Introduction

Aneurysms arising from the posterior communicating artery (PComA) are highly prevalent, as the PComA origin is one of the most frequent sites for intracranial aneurysms(IA) formation, representing approximately 30–35% of all IA and about 50% of all internal carotid artery (ICA) aneurysms (1–4). Individual assessment of IA rupture risk is difficult; a deeper understanding of the factors that precipitate rupture is crucial to advance clinical decision-making.

Morphological parameters and hemodynamics play a crucial role in the growth and rupture risk of IA. However, most research has analyzed aneurysms collectively, rather than focusing on specific locations. Given that aneurysm location is an independent predictor of rupture risk and that hemodynamic features vary based on anatomical structures, further investigation into location-specific aneurysm behavior is essential. In particular, PComA aneurysms exhibit a higher rupture risk compared to those at other locations (5, 6). Therefore, identifying and understanding the risk factors associated with PComA aneurysm rupture remains a crucial research objective.

This urgency is underscored by the high mortality rate (approximately 43%) associated with aneurysmal subarachnoid hemorrhage (SAH) and the substantial risk of rebleeding (60–70%) in untreated cases (7). The development of reliable risk stratification methodologies is imperative to guide clinical decision-making, enabling timely intervention for high-risk aneurysms while avoiding unnecessary procedures in low-risk cases.

Advancements in neuroimaging have significantly improved aneurysm detection; however, accurately predicting aneurysm rupture risk remains a challenge. Risk assessment must account for both uncontrollable factors (e.g., aneurysm size, shape, patient demographics) and modifiable factors (e.g., smoking, hypertension). Educational experience is also associated with health-promoting behaviors, influencing patient outcomes. Studies suggest that individuals with higher educational experience are more likely to seek timely medical care and adhere to treatment recommendations, whereas those with lower educational experience may face challenges in accessing appropriate healthcare services (8).

Previous studies on aneurysm rupture predictors have yielded inconsistent results, and a lack of region-specific data has impeded the development of targeted risk assessment. Given the potential influences of genetic predisposition, lifestyle, and healthcare access, a focused investigation in the Chinese population is essential to address this gap. This study aims to provide a comprehensive analysis of PComA aneurysms in Chinese patients in Western Guangdong by systematically evaluating demographic and clinical characteristics, vessel morphology, and hemodynamic parameters, and rupture status. Our goal is to identify critical risk factors and guide evidence-based management, thereby refining early detection strategies, supporting clinical decision-making, and ultimately improving patient outcomes.

## 2 Materials and methods

### 2.1 Data collection

A retrospective identification of patients diagnosed with PComA aneurysms was conducted using the medical records from the Affiliated Hospital of Guangdong Medical University (Zhanjiang, China), covering the period from January 2020 to December 2024.

The inclusion criteria were as follows: (1) clinical signs and symptoms suggestive of ruptured PComA aneurysms, including headache, eye or neck pain, nausea, vomiting, photophobia, changes in mental status or level of consciousness, epilepsy, dilated pupils, vision alterations, stiff neck, and/or focal neurological deficits, especially oculomotor nerve palsy; (2) verification of PComA aneurysms via head computed tomography angiography (CTA); and (3) access to detailed, complete clinical and imaging data.

The exclusion criteria were as follows: (1) presence of multiple intracranial aneurysms, or specific aneurysm subtypes (e.g., traumatic, dissecting, infectious), or other intracranial vascular abnormalities (e.g., ICA stenosis, cerebral venous sinus thrombosis, or arteriovenous malformations); (2) absence of abnormalities on digital subtraction angiography subsequent to a negative CTA; (3) indeterminate rupture status of PComA aneurysm; (4) insufficient or poor-quality clinical/imaging data that precluded reliable model reconstruction and hemodynamic analysis; and (5) patient or immediate family declined to participate.

Patients with CTA-confirmed PComA aneurysms were divided into two groups for analysis. Specifically, patients with CTA-confirmed PComA aneurysms who also presented with SAH were assigned to the ruptured PComA aneurysm group. In contrast, those with CTA-confirmed PComA aneurysms but no evidence of SAH were categorized into the unruptured PComA aneurysm group. Prior to study initiation, all participants (or their immediate family members, where applicable) were fully informed of the study objectives, procedures, and potential implications. Written informed consent was obtained from each participant or their legal guardian for the use of their de-identified clinical data in this research. This study was conducted in strict accordance with the ethical principles outlined in the Declaration of Helsinki and has received formal approval from the Institutional Review Board of the Affiliated Hospital of Guangdong Medical University.

The sample size was calculated using Power Analysis and Sample Size (PASS) 15.0 (NCSS, LLC, USA) software. To ensure adequate statistical power for detecting significant differences between groups, relevant literature on the morphology and hemodynamics of PComA aneurysms were reviewed to guide the final sample size determination.

**Figure 1:**
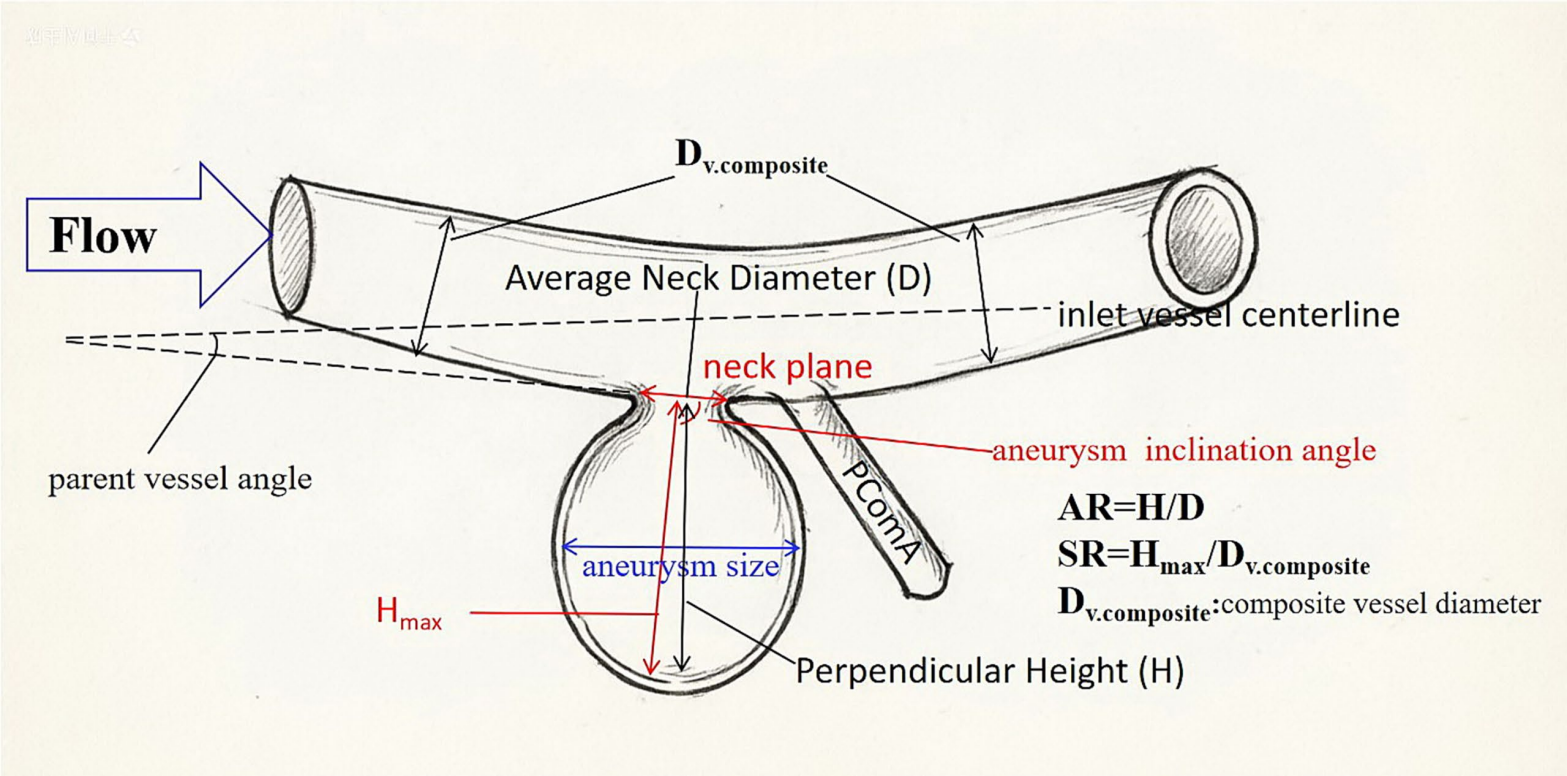
Illustrative examples of measurements for parameters of aneurysm morphology. AR, Aspect ratio; SR, aneurysm-to-vessel size ratio; D_v.composite_, composite vessel diameter.

Collected demographic and clinical data included sex, age, smoking history, alcohol consumption status, admission blood pressure (measured in mmHg), diabetes mellitus status, and educational level. Morphological parameters of the affected blood vessels encompassed aneurysm size (maximum transverse diameter), aneurysm shape feature (regularity or irregularity), aneurysm inclination angle, parent vessel angle, aspect ratio (AR), and aneurysm-to-vessel size ratio (SR) (Figure 1). Hemodynamic parameters evaluated included wall shear stress (WSS) and blood flow velocity (FV) of the aneurysm neck plane. The aneurysm inclination angle is defined as the angle between the connection of the neck centroid to the farthest point on the IA dome, which is a key geometric parameter governing the direct impact force of blood flow from the parent artery onto the aneurysm (9). Parent vessel angle is defined as the angle of the intersection between the inlet vessel centerline and the neck plane (9). Aspect Ratio (AR) is defined as IA height divided by neck diameter (9). Size Ratio (SR) is defined as the ratio of the maximum aneurysm height to the average vessel diameter (9).

To guarantee the independence and objectivity of the assessment, a blind method was implemented. Specifically, the morphological parameters and computational fluid dynamics (CFD)-derived hemodynamic data were evaluated blindly by two associate senior technicians from the CT Room of the Department of Radiology. All evaluations were conducted independently, and any discrepancies were resolved through consensus (or via a third senior technician, if necessary).

### 2.2 Research software and main modeling

A variety of software tools were employed in this study, including Materialise Mimics Innovation Suite Research 19.0 (Materialise Company, Belgium), 3-matic Research 11.0 (Materialise Company, Belgium), Ansys SpaceClaim (ANSYS, Inc., USA), Origin (OriginLab Corporation, USA), ANSYS 2020 R2 Fluent (ANSYS, Inc., USA), Tecplot (Tecplot, Inc., USA), and SPSS Statistics 26.0 (IBM, USA). The analysis was based on original intracranial vascular data, which were exported in DICOM format from the hospital’s Picture Archiving and Communication System (PACS) in the imaging department. The DICOM data were imported into Mimics Research to reconstruct three-dimensional (3D) vascular models and centerlines. Morphological parameters of the affected vessels—such as aneurysm size, shape, inclination angle, parent vessel angle, AR, and SR—were extracted using Materialise Mimics Innovation Suite Research and 3-matic Research. Hemodynamic parameters, including WSS and FV within the aneurysm, were evaluated using Ansys SpaceClaim, Origin, ANSYS 2020 R2 Fluent, and Tecplot.

### 2.3 Statistical analysis

Statistical analyses were conducted using SPSS version 26.0 software. A *p*-value < 0.05 was considered indicative of statistical significance.

## 3 Results

### 3.1 Baseline characteristics of clinical data

**Figure 2:**
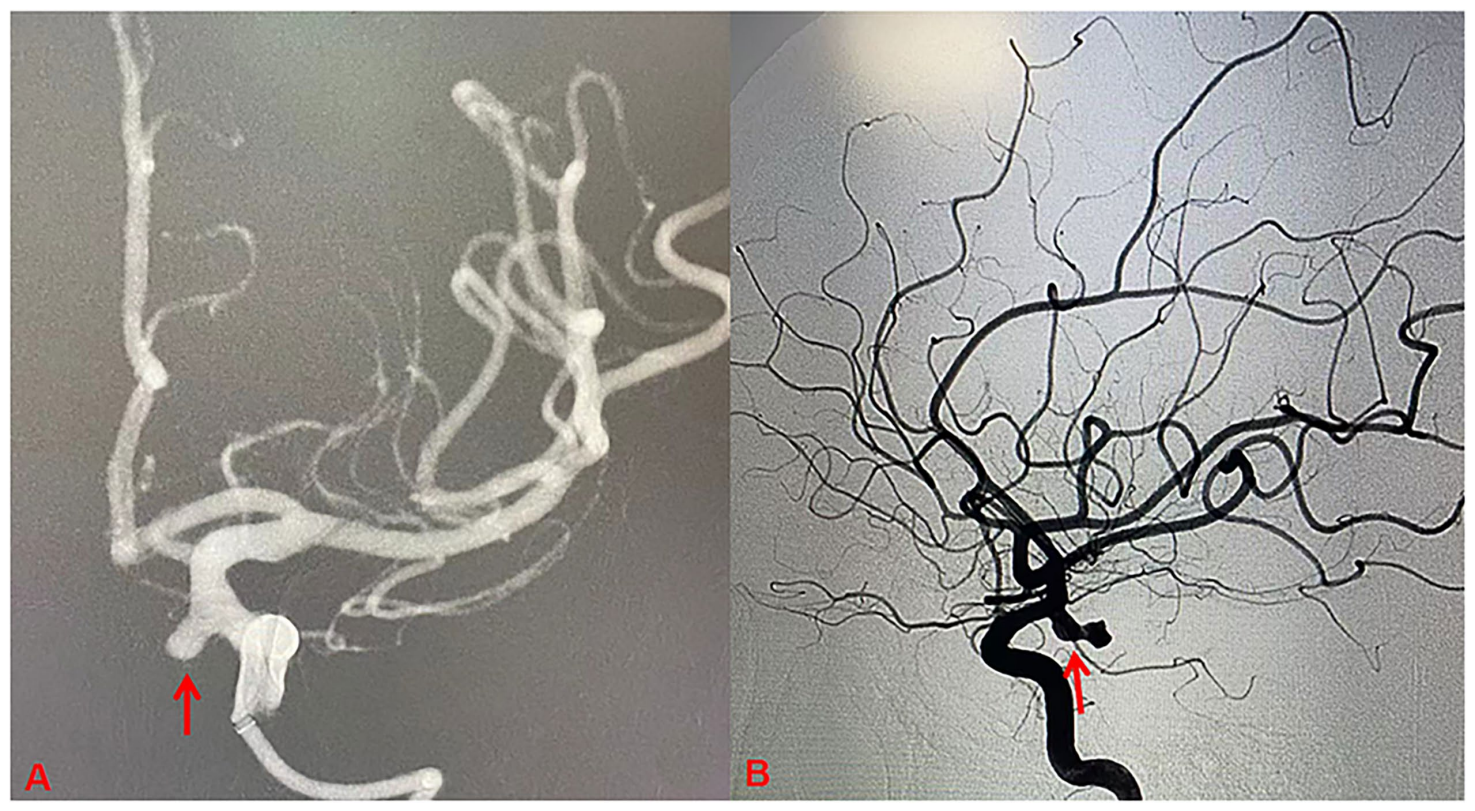
Digital subtraction angiography (DSA) of posterior communicating artery (PComA) aneurysms. **(A)** The PComA aneurysm is highlighted by the red arrow in the DSA roadmap imaging. **(B)** The red arrow indicates a pseudoaneurysm located at the dome of the PComA aneurysm.

The study comprised 224 patients with ruptured PComA aneurysms (50 male, 174 female), who were assigned to the ruptured PComA aneurysm group. Additionally, 138 patients with unruptured PComA aneurysms (30 male, 108 female) were included in the unruptured group (Figure 2). Statistically significant differences were noted between the two groups regarding smoking status, educational experience, blood pressure (mmHg) at the time of admission (*p* < 0.05). The mean systolic pressure was higher in the ruptured PComA aneurysm group than in the unruptured group (164.0 mmHg vs. 150.0 mmHg). Similarly, the mean diastolic pressure was elevated in the ruptured group compared with the unruptured group (97.0 mmHg vs. 86.5 mmHg). Conversely, no meaningful differences were observed in terms of sex, age, alcohol intake, diabetes mellitus status (*p* > 0.05) (Table 1).

**Table 1:** Demographic and clinical characteristics of patients with ruptured and unruptured PComA aneurysms.

Demographic and clinical characteristics	Ruptured group (*n* = 224)	Unruptured group (*n* = 138)	*p*-value
Sex, *n* (%)			>0.05
Male	50 (22.3)	30 (21.7)	
Female	174 (77.7)	108 (78.3)	
Age (median, IQR)	65.0 (54.8–72.00)	63.0 (56.8–70.0)	>0.05
Smoking, *n* (%)			<0.05^*^
Yes	89 (39.7)	40 (29.0)	
No	135 (60.3)	98 (71.0)	
Drinking, *n* (%)			>0.05
Yes	57 (25.4)	35 (25.4)	
No	167 (74.6)	103 (74.6)	
Diabetes, *n* (%)			>0.05
Yes	50 (22.3)	27 (19.6)	
No	174 (77.7)	111 (80.4)	
Educational experience, *n* (%)			<0.05^*^
No education	41 (18.3)	12 (8.7)	
Elementary school	32 (14.3)	13 (9.4)	
Middle school	92 (41.1)	60 (43.5)	
College	49 (21.9)	46 (33.3)	
Graduate student	10 (4.5)	7 (5.1)	
Systolic pressure (mmHg, median, IQR)	164.0 (153.8–177.3)	150.0 (138.8–158.0)	<0.05^*^
Diastolic pressure (mmHg, median, IQR)	97.0 (92.8–105.0)	86.5 (82.0–91.0)	<0.05^*^

Continuous variables are presented as mean ± standard deviation (SD) for normally distributed data and as median (interquartile range) for data with a non-normal distribution. Categorical variables are expressed as counts and percentages. Statistical comparisons were conducted using the Wilcoxon rank-sum test, t-test, or chi-squared test as appropriate. *p < 0.05 was considered indicative of statistical significance.

### 3.2 Blood vessel morphology characteristics

Significant intergroup differences were identified with respect to the aneurysm size, shape feature, aneurysm inclination angle, SR (*p* < 0.05) (Table 2). Aneurysm size, irregular shapes, aneurysm inclination angle and SR in the ruptured PComA aneurysm group were higher than that in the unruptured PComA aneurysm group. Conversely, vessel angle, and AR did not exhibit significant differences (*p* > 0.05).

**Table 2:** Blood vessel morphology characteristics of patients with ruptured and unruptured PComA aneurysms.

Morphology characteristics	Ruptured group (*n* = 224)	Unruptured group (*n* = 138)	*p*-value
Aneurysm size (mm, median, IQR)	4.6 (3.8–5.3)	2.9 (2.5–3.3)	<0.05^*^
Shape feature, *n* (%)			<0.05^*^
Regular shapes	73	87	
Irregular shapes	151	51	
Aneurysm inclination angle (median, IQR)	112.3 (104.7–120.8)	84.75 (77.13–92.33)	<0.05^*^
Vessel angle (median, IQR)	26.6 (22.5–30.6)	26.40 (21.88–30.25)	>0.05
Aspect ratio (median, IQR)	1.42 (1.31–1.52)	1.41 (1.32–1.49)	>0.05
Size ratio (median, IQR)	1.61 (1.45–1.78)	1.24 (1.09–1.31)	<0.05^*^

Continuous variables are presented as mean ± standard deviation (SD) for normally distributed data and as median (interquartile range) for data with a non-normal distribution. Categorical variables are expressed as counts and percentages. Statistical comparisons were conducted using the Wilcoxon rank-sum test, t-test, or chi-squared test as appropriate. ^*^p < 0.05 was considered indicative of statistical significance.

### 3.3 Hemodynamic parameters of blood vessel characteristics

**Figure 3:**
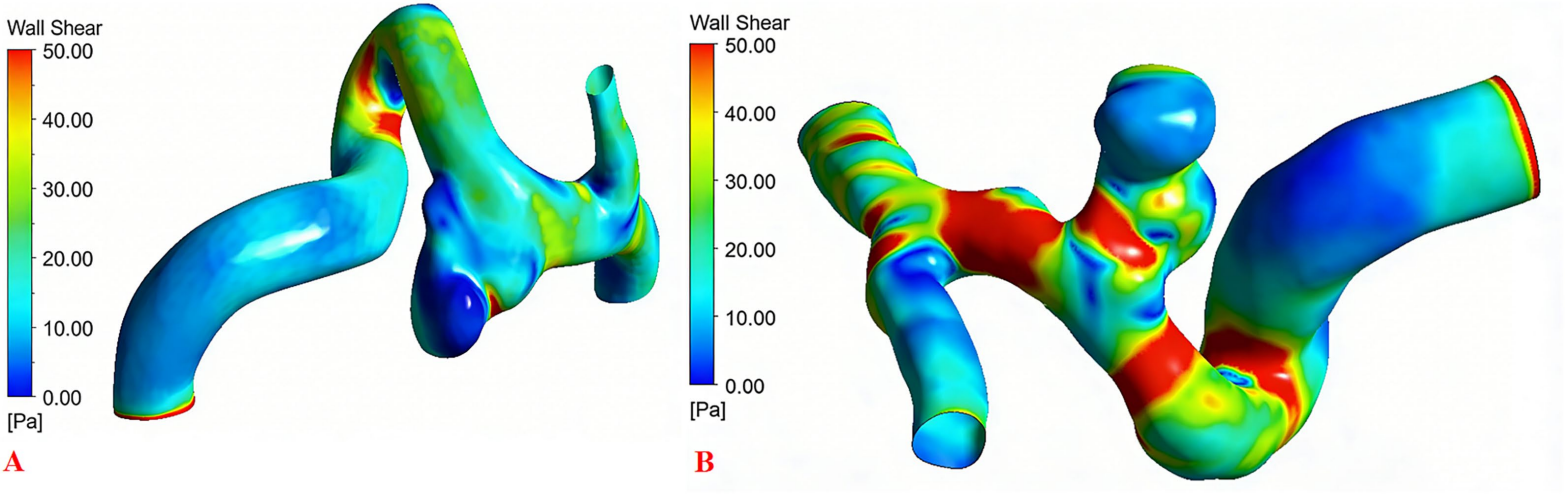
**(A)** WSS in ruptured PComA aneurysms group. **(B)** WSS in unruptured PComA aneurysms group. WSS, wall shear stress.

Significant differences in hemodynamic parameters were observed between the two groups. WSS and FV was significantly lower in the ruptured PComA aneurysm group compared to the unruptured group (*p* < 0.05) (Table 3; Figure 3).

**Table 3:** Hemodynamic parameters of blood vessel characteristics of patients with ruptured and unruptured PComA aneurysms.

Hemodynamic parameters	Ruptured group (*n* = 224)	Unruptured group (*n* = 138)	*p*-value
WSS (Pa, median, IQR)	7.12 (6.51–7.78)	8.33 (7.36–9.06)	<0.05*
flow velocity(m/s, median, IQR)	0.07 (0.06–0.08)	0.24 (0.19–0.30)	<0.05*

Continuous variables are presented as mean±standard deviation (SD) for normally distributed data and as median (interquartile range) for data with a non-normal distribution. Categorical variables are expressed as counts and percentages. Statistical comparisons were conducted using the Wilcoxon rank-sum test, t-test, or chi-squared test as appropriate. *p < 0.05 was considered indicative of statistical significance. WSS, wall shear stress.

## 4 Discussion

Rupture of IA are a major cause of spontaneous SAH and are associated with high severe neurological impairment or even death (10). Identifying risk factors for aneurysm rupture and implementing appropriate preventive measures may help reduce or prevent SAH. However, consensus on these risk factors remains elusive. Anatomical location is a key determinant. Aneurysms located in the PComA exhibit a higher rupture propensity compared with those at other intracranial sites (11–13). Patients with PComA aneurysms are particularly at risk, highlighting the need for careful risk stratification and benefit–risk assessment when considering prophylactic intervention for unruptured PComA aneurysms.

Previous research has demonstrated that smoking and hypertension are significant risk factors for the rupture of intracranial aneurysms (14–16). In this study, significant differences were noted between the ruptured and unruptured PComA aneurysm groups concerning smoking status, educational experience, and systolic and diastolic blood pressure (mmHg) at the time of admission (*p* < 0.05). These findings are consistent with the known risk profile, implicating smoking and elevated blood pressure in the rupture risk of PComA aneurysms.

Aneurysm size refers to the maximum perpendicular height (i.e., the greatest distance measured perpendicularly from the neck plane to the dome) (9, 17). Shape feature is based on the morphological classification of IA on three-dimensional rotational angiography (3D-RA). Regular aneurysms exhibit a smooth and uniformly curved contour, while irregular aneurysms are defined by the presence of focal irregularities at the domes, including blebs (small blister-like protrusions) or secondary sacs, or by a dome that is distinctly bilobed or multilobed (18–21). In this study, significant differences were observed in aneurysm size, morphological characteristics, aneurysm inclination angle, and SR (all *p* < 0.05). Unlike the findings of previous studies, our results suggest that alterations in vessel geometry may contribute to the rupture risk of PComA aneurysms (22).

As a measure of the tangential frictional force exerted by flowing blood on the endothelium, WSS is a critical hemodynamic factor in aneurysm progression and rupture. The FV at the neck plane is another critical hemodynamic metric for intracranial aneurysms, representing the inflow kinetics into the aneurysm sac. Its magnitude and pattern are closely associated with WSS distribution and are believed to influence aneurysm initiation, progression, and rupture risk. WSS and FV were significantly lower in the ruptured PComA aneurysm group compared to the unruptured group in this study (*p* < 0.05). Previous studies indicated that lower WSS and FV might be related to the high rupture risk of PComA aneurysms, which support our findings (23–26).

In summary, this study highlights the importance of identifying and managing risk factors for PComA aneurysm rupture. Targeted prevention and management are crucial to reducing the risk of hemorrhage. Routine screening with CTA or MRA is advised for high-risk individuals — especially hypertensive smokers with low educational experience. For unruptured PComA aneurysms with high-risk morphological or hemodynamic features, surgical intervention should be considered. When surgery is not immediately indicated, close monitoring with serial imaging is recommended, particularly if modifiable risk factors such as hypertension or smoking are present. If imaging follow-up shows progression in morphology or hemodynamics despite controlled blood pressure and smoking cessation, surgical management is strongly warranted in medical healthcare services.

There are some limitations in this study. First, the retrospective design of this study means that the collection of data is potential biased, the observed differences in morphological and hemodynamic parameters between ruptured and unruptured aneurysms cannot be distinguished as causes or consequences of rupture, and therefore causality cannot be established. Second, the single-center origin and relatively limited sample size may restrict the generalizability of the findings. In particular, because the cohort was recruited solely from the Affiliated Hospital of Guangdong Medical University, the results may be influenced by local clinical practices, patient demographics, and referral patterns. These factors could affect the external validity of the study and limit the applicability of any derived risk-stratification tools or clinical decisions. Third, most hemodynamic studies on rupture risk have compared postruptured and unruptured IAs (24, 25, 27). However, once an aneurysm ruptures, its morphology and hemodynamics may change, which could reduce the accuracy of such comparisons and limit the clinical applicability of the derived parameters for rupture assessment (28). Therefore, focusing on unruptured IAs at high risk of rupture may yield more reliable and meaningful insights (24). Finally, the inclusion of participants exclusively of Chinese ethnicity from Western Guangdong in southern China may limit the generalizability of the findings, as results could be influenced by specific genetic, cultural, or regional factors not present in other populations.

## 5 Conclusion

Given the heightened risk among hypertensive smokers who have not received a good education, routine screening via CTA or MRA is recommended. The unruptured PComA aneurysm with larger aneurysm size, irregular shapes, aneurysm inclination angle and SR and lower WSS and FV may have a high-risk to be ruptured in the future. Surgical intervention should be prioritized for high-risk aneurysms, while close imaging follow-up is advised for patients without immediate surgical indications. If morphological or hemodynamic changes are detected over time, surgical treatment should be strongly considered. These findings contribute to improved prevention, management, and control of PComA aneurysm rupture, as well as in policy development to institutionalize these strategies.

The relative contributions of morphology and hemodynamics, their possible correlations, and the ability of these markers to predict rupture risks need to be assessed in larger prospective studies, multi-center, and ethnically diverse cohorts that include follow-ups of patients with unruptured PComA aneurysms.

## Data Availability

The original contributions presented in the study are included in the article/supplementary material, further inquiries can be directed to the corresponding authors.
